# Surface Activation and Characterization of Basalt Fiber by Plasma Treatment and Its Interfacial Adhesion with Epoxy

**DOI:** 10.3390/polym16223181

**Published:** 2024-11-15

**Authors:** Guowan Guo, Zhongjia Yang, Mingjun Cai, Shuhan Wang, Lei Jiang

**Affiliations:** 1 Laboratory of Bio-Inspired Smart Interfacial Science and Technology of Ministry of Education, School of Chemistry, Beihang University, Beijing 100191, China; 2 Sichuan Basalt Fiber New Material Research Institute (Innovation Center), Guang’an 638500, China; 3 Key Laboratory of Bio-Inspired Materials and Interfacial Science, Technical Institute of Physics and Chemistry, Chinese Academy of Sciences, Beijing 100191, China

**Keywords:** basalt fiber, epoxy, interfacial adhesion, surface activation, plasma

## Abstract

The weakness of the fiber–matrix interface restricts the practical application of basalt fiber (BF) as a reinforcing material. In order to improve the interfacial adhesion between the BF and epoxy matrix, surface activation of the BF was carried out using low-pressure O_2_ and H_2_-Ar plasma under various conditions. The interfacial shear strength (IFSS), evaluated by a micro-droplet de-bonding test, was adopted to demonstrate the bonding effects at the BF/epoxy interphase. Compared to bare BF, the IFSS between the modified fibers and epoxy matrix was efficiently improved with an increment of 38.4% and 14.4% for O_2_ plasma and H_2_-Ar plasma treatment, respectively. Scanning Electron Microscope (SEM) and Atomic Force Microscopy (AFM) analysis indicated that H_2_-Ar plasma-treated BF had a much rougher and more rugged surface than O_2_ plasma-treated samples. X-ray Photoelectron Spectroscopy (XPS) and surface energy results revealed that O_2_ plasma activation could effectively increase the content of oxygenous groups on the BF surface, thus resulting in a higher total surface energy value. Based on the results, O_2_ plasma modification at a power of 200 W and pressure of 80 Pa for 0.5 min was considered to be the most favorable condition for the surface activation of BF.

## 1. Introduction

Among all high-performance fibers, basalt fiber (BF) boasts the lowest energy consumption for production compared to carbon fiber, aramid fiber, or ultra-high-molecular weight polyethylene fiber. In addition, during the manufacturing process, no chemical additives or solvents are added, and no hazardous components are generated owing to the thermal stability of the raw basalt rocks, which hardly contain any unstable ingredients after volcanic eruption [1]. Thus, BF is a typically resource-conserving and environmentally friendly fiber, aligning with the trend towards energy conservation, emission reduction, and the development of a low-carbon economy [2]. Owing to its outstanding mechanical performance, heat insulation, and sound absorption, as well as resistance to chemical corrosion, nuclear radiation, and high-temperature failure [3,4,5], BF can be used in many fields, such as the aerospace, wind power generation, ocean engineering, nuclear, and automotive industries. Usually, BF acts as the reinforcing material to prepare polymer- or cement-based composites for the above applications, but the fiber–matrix interface of BF-reinforced composites is weak because of the low surface energy, small specific surface area, and high chemical inertness of BF [6]. Therefore, surface modification of BF is quite necessary for its practical application.

In general, the surface modification methods of BF include chemical etching, sizing agent coating, and nanomaterial engineering. Among these embellishing processes, chemical etching could indeed improve the interfacial shear strength (IFSS) between BF and the matrix. However, it is also accompanied by a deterioration in the tensile strength of the fibers [7]. The sizing agent coating method mainly focuses on the purposes of monofilament bundling and further woven knitting [8]. Nanomaterial modification would obviously improve the bonding strength of the fiber–matrix interface by increasing the surface roughness of BF on the micro-scale. Nevertheless, this tailoring technic always needs complex processes, such as electrophoretic deposition [9,10] and chemical vapor deposition (CVD) [11,12], to avoid the agglomeration of nanomaterials.

Plasma, a highly ionized gas consisting of ions, radicals, excited molecules, and free electrons, has been widely used in industrial manufacturing and environmental control [13,14]. Considering that the plasma affects only a shallow layer of the materials’ surface at nano- and micro-metric scales [15,16], the surface properties of the substrates, such as the topography, wettability, roughness, and adsorption of biomolecules [17,18] would be altered while the bulk properties, including their physical and mechanical properties, might be maintained intact. Hence, plasma treatment seems to be a perfect technique for the surface modification of BF since natural strength is critical for BF when taking on the role of a reinforcing material. Many researchers have paid attention to the plasma modification of BF. The variation in fiber strength, surface properties, and the interlaminar fracture properties of BF treated by an atmospheric-pressure plasma of oxygen, argon, hydrogen, and a mixture of gases was reported by some papers [19,20,21]. Recently, the plasma polymerization process has attracted the interest of researchers and has already been used for the surface modification of BF [22,23,24,25]. However, this technique requires additional chemicals, such as plasma gas, as well as a long treatment duration, thus minimizing the convenience and practicability of plasma methods. To the best of the authors’ knowledge, little research has been carried out to study the effects of low-pressure plasma treatment on the adhesion between BF and the epoxy matrix.

The interfacial adhesion property of fiber-reinforced composites can be characterized by a shear test performed on actual composite specimens. Although this test provides valuable information about the overall or macroscopic properties at the fiber/matrix interphase, the effects of the specimen’s geometry, fiber volume fraction, and fiber aspect ratio cannot be avoided. In recent years, direct measurement of strength in a monofiber/matrix system was developed to evaluate the interfacial bonding between the fiber and matrix. Cherevinskiy et al. [26] investigated the strength in a unit cell of a carbon monofiber/cured epoxy matrix before and after treatment in oxygen discharge plasma. Tian et al. [27] applied the electrospinning method to prepare polyacrylonitrile nanofiber-sheathed BF, and a monofiber pull-out test was used to assess the interfacial properties between the BF and epoxy. Zhang et al. [28] obtained an enhanced BF/epoxy interface by chemical grafting aliphatic chains onto the BF surface, in which the micro-droplet de-bonding method was also adopted.

The aim of this paper is to investigate the effects of low-pressure plasma modification on the interfacial adhesion property between BF and the epoxy matrix. The interfacial shear strength (IFSS), evaluated by the micro-droplet de-bonding test, was adopted to demonstrate the bonding effects. A comprehensive set of analytical methods, including Scanning Electron Microscope (SEM), Atomic Force Microscopy (AFM), X-ray Photoelectron Spectroscopy (XPS), and contact angle measurement, was conducted to clarify the enhancing mechanism of plasma activation.

## 2. Materials and Methods

### 2.1. Materials

BF without sizing was supplied by Sichuan Basalt Fiber New Material Research Institute (Innovation Center) (Guang’an, China). Ultrasonically washing by acetone was implemented to clear the impurities on the BF’s surface before use. High-purity oxygen gas (99.999%) and a hydrogen–argon gas mixture (5%:95%) were purchased from AST Tech Co., Ltd. (Chongqing, China). Epoxy resin (AIRSTONE 760E) and the curing agent (AIRSTONE 766H) were obtained from Olin Corporation (Clayton, MO, USA). Formamide and diiodomethane were procured from Macklin Biochemical Technology Co., Ltd. (Shanghai, China).

### 2.2. Surface Activation of Basalt Fiber

BF yarns were separated into single fibers and immobilized to a rigid paper frame before plasma treatment. The surface activation process was conducted using a HX-30-QXJ low-pressure plasma generator (Hengxin Semiconductor Equipment Co., Ltd., Dongguan, China) with a frequency of 13.56 MHz and using O_2_ and H_2_-Ar as the source gas, respectively. To obtain the optimal parameters, BF samples treated with varying powers (100, 200, and 300 W), pressures (20, 40, 60, and 80 Pa), and times (0.5, 1, 3, 5, and 7 min) were fabricated. Then, the fiber samples were immediately placed in sterile zip bags to reduce the risk of contamination.

### 2.3. Measurement of Interfacial Shear Strength

The IFSS between BF and epoxy matrix was estimated via a micro-droplet de-bonding test on a UTM 2201 universal tensile testing machine (SUNS, Shenzhen, China) equipped with a force sensor with a nominal load of 5 N. A microscope system composed of a zoom 6000 lens (Navitar, Rochester, NY, USA) and a DMK33UX264 camera (The Imaging Source, Bremen, Germany) was applied to observe the micro-droplet in real-time. The embedded length of fiber in the epoxy droplet was measured using IC Capture (version number: 2.5.1557.4007) software. When conducting the pull-out test, a knife-like clamp was adapted to hold the droplet, and the loading rate was set at 0.005 mm/s. To prepare samples for the micro-debonding test, the homogeneous mixture of 760E epoxy and a 766H curing agent with a mass ratio of 100:32 was deposited on a single fiber surface to form resin droplets. This step should be completed within 2 h after surface activation of the basalt fiber. Subsequently, the single fiber/epoxy system was cured at 150 °C for 4 h. The IFSS value is calculated by the following equation:

IFSS = F_max_/πdl (1)

where F_max_ denotes the pullout force, d is the diameter of the basalt fiber, and l is the embedded length of fiber in the epoxy droplet.

### 2.4. Characterizations

The surface morphologies of BF were analyzed on a Sigma 300 Scanning Electron Microscope (ZEISS, Oberkochen, Germany) and Dimension Icon Atomic Force Microscopy (Bruker, Billerica, MD, USA). When calculating the surface roughness, the scanning region was fixed at 1 × 1 μm. The chemical compositions of BF were evaluated through K-Alpha X-ray Photoelectron Spectroscopy (Thermo Scientific, Waltham, MA, USA). Si2p and O1s spectra were treated by virtue of the Avantage (version number: 5.9931) software, and a binding energy of 284.8 eV was taken as a reference for calibration. The contact angles of basalt glass with water, formamide, and diiodomethane were measured by a DSA25S Contact Angle analyzer (KRUSS, Hamburg, Germany) and the surface energy was acquired by calculations based on the OWRK method [29]. The basalt glass was gradually polished by varying meshes of sandpaper to obtain an extremely smooth surface before testing.

The raw BF, BF treated by O_2_ plasma at 20 and 80 Pa (abbreviated as BF_(OP-20Pa)_ and BF_(OP-80Pa)_), and BF treated by H_2_-Ar plasma for 1 and 5 min (abbreviated as BF_(HAP-1min)_ and BF_(HAP-5min)_) were characterized by the above analytical methods.

## 3. Results and Discussion

### 3.1. Interfacial Shear Strength Between Basalt Fibers and Epoxy

Generally, the glow discharging power influences the speed and kinetic energy of plasma, the pressure determines the plasma density, and the exposure time affects the collision probabilities of plasma with BF, thereby significantly impacting the interactions between the plasma and fiber surface. Appropriate plasma energy, density, and processing duration can yield better surface activation of BF, whereas excessive treatment techniques may cause fiber surface damage, thus deteriorating the natural strength of BF. In order to obtain the optimal parameters for BF’s surface activation, the effects of treatment power (100–300 W), pressure (20–80 Pa), and time (0.5–7 min) on the IFSS between the fiber and epoxy were investigated.

**Figure 1:**
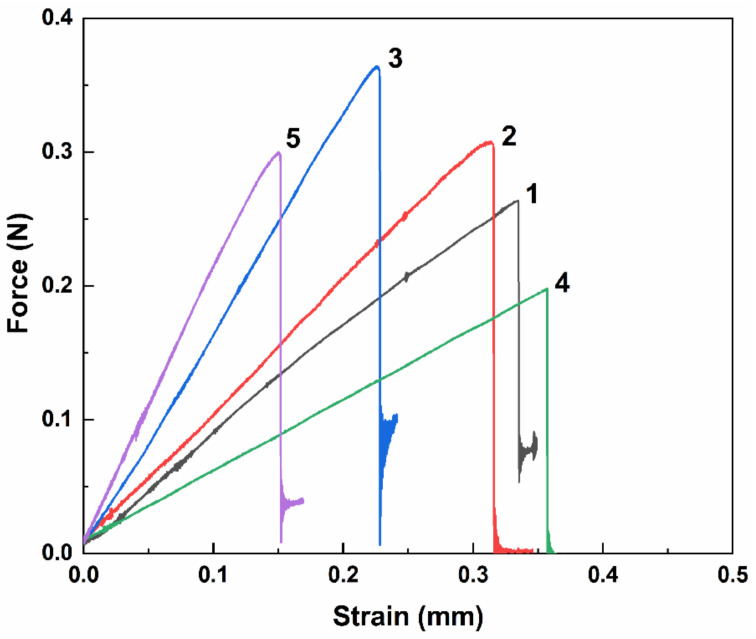
Typical force–strain curves of BF pulling out from epoxy: (1) neat BF, (2) BF treated with O_2_ plasma at 20 Pa (BF_(OP-20Pa)_), (3) BF treated with O_2_ plasma at 80 Pa (BF_(OP-80Pa)_), (4) BF treated with H_2_-Ar plasma for 1 min (BF_(HAP-1min)_), and (5) BF treated with H_2_-Ar plasma for 5 min (BF_(HAP-5min)_).

From the data (Table 1), it can be seen that the IFSS between BF and the epoxy increased with the amplification of treatment power from 100 to 200 W when O_2_ was used as the plasma resource. Further power increases resulted in negligible growth of the IFSS, which indicates that 200 W of power is sufficient for O_2_ plasma-induced reactions. Treatment pressure ranging from 20–80 Pa dramatically affected the interfacial adhesion of the BF and epoxy, where 80 Pa of pressure presented the highest IFSS of 35.08 ± 1.96 MPa, with a 38.4% increment in comparison to that of neat BF. Since 80 Pa is the maximum pressure that can be adjusted for our plasma generator, no further increases in pressure were explored. The IFSS value showed no significant differences among the treatment time of 0.5–5 min. For H_2_-Ar plasma treatment, variations in power and pressure showed similar changing trends on the IFSS between BF and epoxy as seen with O_2_ plasma. In addition, treatment time displayed a prominent impact on IFSS values. It was interesting to observe from the data that 1–3 min of H_2_-Ar plasma treatment decreased the IFSS to 19.10–22.02 MPa, which may be attributed to the loss of functional groups on the BF surface. With the extension of treatment time, the IFSS reached a higher value of 28.99 ± 2.36 MPa at a time of 5 min, with augmentation of 14.4% related to blank BF. This may be explained by the fact that the enhancement of surface roughness compensates for the loss of functional groups. For reference, some typical force-strain curves of BF pulling out from epoxy were presented in Figure 1. Comparing our results to other surface modification methods, we found that a 38.4% increment in the IFSS between the BF and epoxy was not conspicuous with respect to the growth of 31.3–47.0% published in some studies [27,28,30]. However, it is definitely the most efficient way to complete the activation process within 30 s. Moreover, plasma activation can be applied as the pre-treatment technique combined with further modification, such as sizing agent coating and nanomaterial engineering.

### 3.2. Surface Morphologies of Basalt Fibers

**Figure 2:**
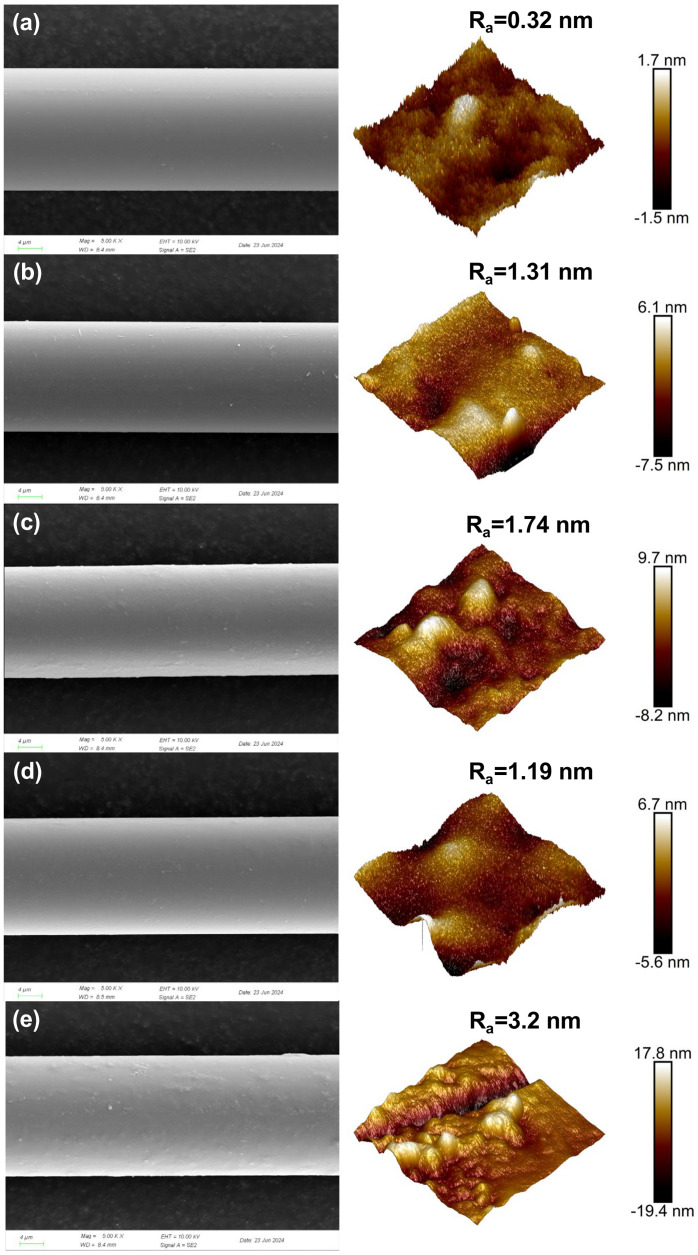
Scanning Electron Microscope (SEM) and Atomic Force Microscopy (AFM) morphologies of neat BF (**a**), BF_(OP-20Pa)_ (**b**), BF_(OP-80Pa)_ (**c**), BF_(HAP-1min)_ (**d**), and BF_(HAP-5min)_ (**e**).

The surface morphologies of BFs treated with O_2_ plasma (20 and 80 Pa) and H_2_-Ar plasma (1 and 5 min) were illustrated via SEM, respectively. As shown in Figure 2a, neat BF presented a relatively slippery and slightly striped surface. After the activation of O_2_ plasma at 20 Pa, more speckles and stripes were discovered on the BF surface (Figure 2b). With the increment of working pressure to 80 Pa, the surface morphologies of BF became increasingly complex with more notable stripes and some dimly visible protuberances (Figure 2c). Meanwhile, similar differences were also observed in the case of H_2_-Ar plasma modification (Figure 2d,e), but the BF surfaces were much rougher and more rugged than those of O_2_ plasma-treated samples. To directly verify the roughness variation of BFs, AFM analysis was employed and the surface roughness—the arithmetic mean of the absolute height difference between the testing region and central panel (Ra)—of fibers was calculated. From the data, the surface roughness of BF_(OP-20Pa)_ and BF_(OP-80Pa)_ increased from 0.32 nm to 1.31 nm and 1.74 nm, respectively. As for BF_(HAP-5min)_, it reached the highest surface roughness of 3.2 nm, which is in good agreement with the SEM images. The improved roughening effects of H_2_-Ar plasma may be ascribed to the outstanding bombing ability of argon, which has a greater atomic radius, or the exposure of metal and silicon elements caused by the reductive reaction of hydrogen. This roughness characteristic endows BF with superior surface interactions with the wetted epoxy matrix, as well as mechanical interlocking.

### 3.3. Surface Chemical Characteristics of Basalt Fibers

**Figure 3:**
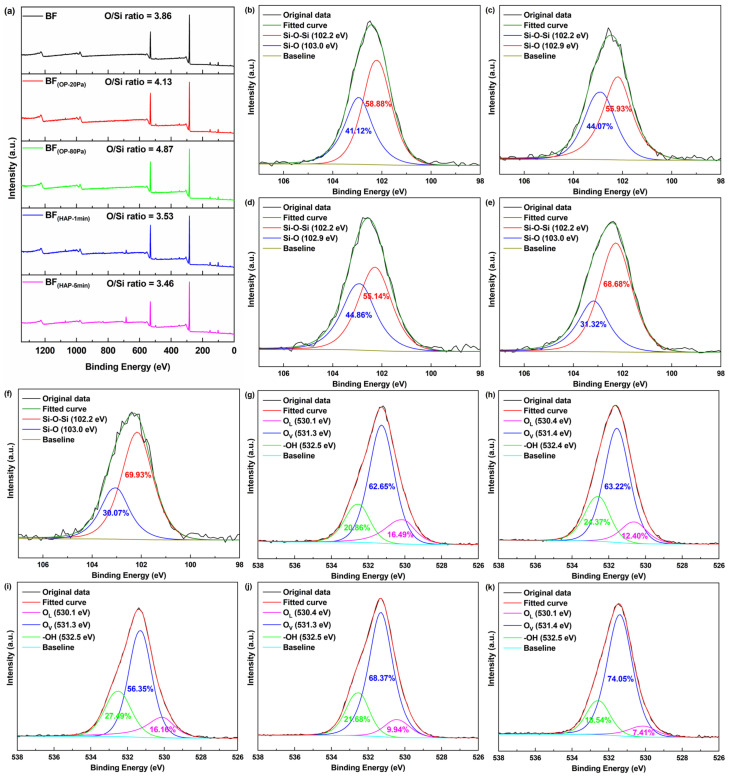
X-ray Photoelectron Spectroscopy (XPS) survey spectra of basalt fibers (**a**); Si2p spectra of neat BF (**b**), BF_(OP-20Pa)_ (**c**), BF_(OP-80Pa)_ (**d**), BF_(HAP-1min)_ (**e**), and BF_(HAP-5min)_ (**f**); O1s spectra of BF (**g**), BF_(OP-20Pa)_ (**h**), BF_(OP-80Pa)_ (**i**), BF_(HAP-1min)_ (**j**), and BF_(HAP-5min)_ (**k**).

The XPS survey spectra of BFs (Figure 3a) revealed no large differences in chemical components before and after surface activation except that fluorine was detected in H_2_-Ar plasma-treated fibers, which may be attributed to the impurities introduced by the H_2_-Ar gas mixture. In order to explore the variation of oxygenous groups in each sample, the ratio of oxygen and silicon (O/Si) was calculated. According to the data, O_2_ plasma activation can efficiently improve the content of oxygenous groups in BFs, where BF_(OP-80Pa)_ had the highest O/Si ratio of 4.87 compared to bare BF (3.86). Meanwhile, H_2_-Ar plasma treatment decreased the O/Si ratio to 3.53 for BF_(HAP-1min)_ and 3.46 for BF_(HAP-5min)_, respectively, due to the reductive reaction of hydrogen.

The high-resolution spectra and deconvolution results of Si2p and O1s are shown in Figure 3b–k. From Si2p observation of the BF (Figure 3b), the two peaks at 102.2 and 103.0 eV were attributed to the Si-O-Si structure and the Si-O bond, respectively [31,32]. The Si-O bond content of O_2_ plasma-treated samples increased to 44.07–44.86% compared to raw BF (41.12%), while in the case of H_2_-Ar plasma, the value was reduced to 30.07–31.32%. The fitting curves of O1s for BF samples presented three peaks at ~530.1, ~531.3, and ~532.5 eV, which correspond to lattice oxygen (O_L_), vacancy oxygen (O_V_), and hydroxyls (-OH), respectively [33,34,35]. The content of -OH of O1s was calculated to be 24.37% for BF_(OP-20Pa)_ and 27.49% for BF_(OP-80Pa)_, which are higher than for neat BF (20.86%). As for H_2_-Ar plasma-treated BFs, the content of -OH showed a slightly fluctuating change, while the percentage of O_L_ decreased to 7.41–9.94%, with O_V_ content increasing to 68.37–74.05% in comparison to bare BF (16.49% of O_L_, 62.65% of O_V_). The results divulge that the oxygenous groups newly introduced by O_2_ plasma activation are mainly composed of Si-OH, which would improve the surface energy of BF materials. In addition, the loss of oxygen elements by H_2_-Ar plasma treatment could be attributed to the destruction of Si-O crystal structures caused by the reductive reaction of hydrogen.

### 3.4. Surface Energy of Basalt Glasses

**Figure 4:**
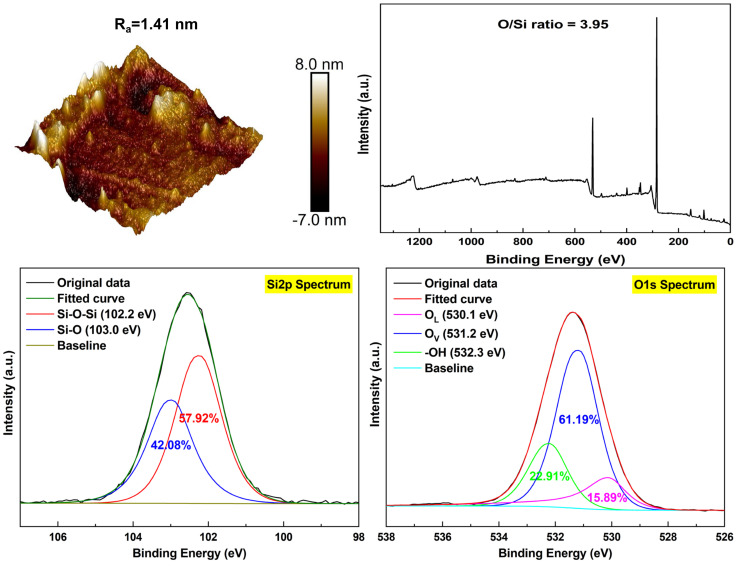
AFM morphologies and XPS spectra of basalt glass (BG).

**Figure 5:**
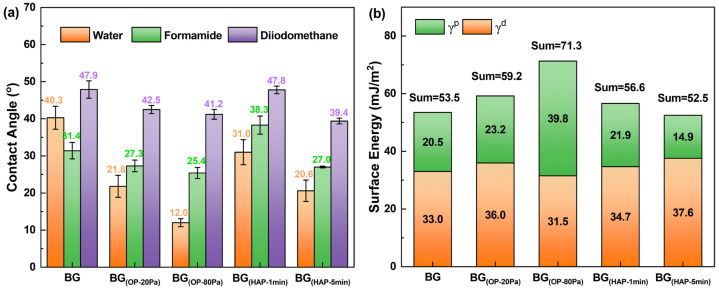
Contact angle of BG with water, diiodomethane, and formamide (**a**); surface energy of BG (**b**).

Since the low-viscosity droplets can hardly be located on the single basalt fiber, a basalt glass (BG) prepared by a similar manufacturing process as BF was used instead of the fiber for contact angle and surface energy testing. The surface roughness and chemical compositions of the BG were analyzed by AFM and XPS (Figure 4). It was clear from the data that BG exhibited similar surface characteristics and chemical components with BF. As shown in Figure 5a, the contact angles of neat BG with deionized water (polar solvent) and formamide (partially polar solvent) were 40.3 ± 3.1° and 31.4 ± 2.2°, while after the surface activation of O_2_ plasma, the wetting angles exhibited a trend of gradual decline to the lowest values of 12.0 ± 1.1° and 25.4 ± 1.5°, respectively, for BG_(OP-80Pa)_ (BG treated by O_2_ plasma at 80 Pa). Such variations directly led to a 94.1% increment in the polar part of surface energy (γ^p^) from 20.5 mJ/m^2^ for BG to 39.8 mJ/m^2^ for BG_(OP-80Pa)_ (Figure 5b). This could be attributed to the increasing amount of polar functional groups (oxygenous groups) on the BG surface as elucidated by the XPS analysis of BFs. By contrast, H_2_-Ar plasma treatment reduced the γ^p^ value of BG samples due to the decline of oxygenous groups caused by the reductive reaction of hydrogen. However, the contact angles of BG_(HAP-1min)_ and BG_(HAP-5min)_ (BG treated by H_2_-Ar plasma for 1 and 5 min) with water still descended because of the enhancement of fiber surface roughness, which could be verified by the greater values (34.7–37.6 mJ/m^2^) in the dispersive part of surface energy (γ^d^) compared to virgin BG (33.0 mJ/m^2^) [36,37]. It was clear from the data that BG_(OP-80Pa)_ had the highest total surface energy of 71.3 mJ/m^2^, while BG_(HAP-5min)_ showed the lowest value of 52.5 mJ/m^2^. In most cases, higher surface energy results in better wetting behavior [38,39], which is especially beneficial for improving the interfacial adhesion of the BF with epoxy resin.

### 3.5. Enhancing Mechanism

**Figure 6:**
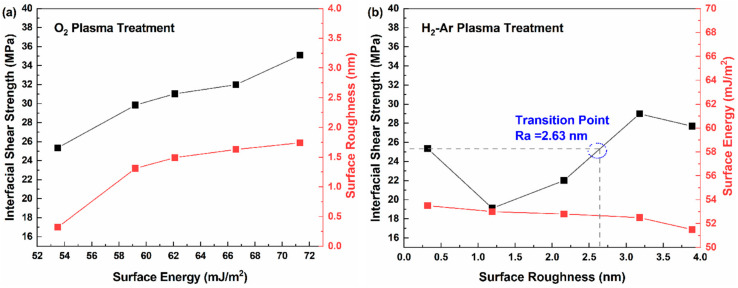
Correlation between the surface energy, surface roughness of BF, and IFSS of BF/epoxy with O_2_ plasma treatment (**a**) and H_2_-Ar plasma treatment (**b**).

In order to demonstrate the enhancing mechanism of plasma activation on the interfacial adhesion between the BF and epoxy, the correlation between surface energy, surface roughness, and IFSS is observed in Figure 6. For O_2_ plasma treatment (Figure 6a), both surface energy and surface roughness were directly proportional to the IFSS. With the increasing surface energy and surface roughness of O_2_ plasma treatment, the BF/epoxy interfacial combination was greatly enhanced due to the improved wetting behavior of the BF and amplified micro-mechanical interlocking between the fiber and matrix. In the case of H_2_-Ar plasma (Figure 6b), with the gradual decline in the surface energy of the BF, the IFSS value presented a falling then rising trend, which was attributed to the reinforcement of surface roughness. When Ra reached a value of 2.63 nm, the IFSS of the modified BF surpassed that of the neat BF, meaning that the growing surface roughness finally overcame the negative effects of the decreasing surface energy. Thus, it can be seen that the enhancing mechanism of plasma activation is mainly ascribed to the synergetic effects of surface energy and surface roughness.

## 4. Conclusions

The interfacial adhesion between the BF and epoxy matrix was efficiently improved by the surface activation of the BF with O_2_ or H_2_-Ar plasma. Under the optimal activation conditions, in comparison with neat BF, O_2_ plasma treatment showed a higher IFSS increment of 38.4% than the H_2_-Ar plasma method (14.4%). Analytical results reveal that the enhancement of the interfacial properties of BF/epoxy obtained by O_2_ plasma activation is mainly ascribed to the improved surface energy of BF, which benefits from the increase in oxygenous groups. For H_2_-Ar plasma treatment, the falling then rising trend of IFSS values is the competitive result of the loss of functional groups and the enhancement of surface roughness. Thus, the reinforcing mechanism of plasma activation is mainly attributed to the synergetic effects of surface energy and surface roughness, which correspond to the wetting behavior of the BF and micro-mechanical interlocking between the fiber and matrix, respectively. Based on the results, O_2_ plasma modification at a power of 200 W and a pressure of 80 Pa for 0.5 min was considered to be the most favorable conditions for the surface activation of BF. This technique can be applied as a pre-treatment method combined with further modification, such as sizing agent coating and nanomaterial engineering.

## Figures and Tables

**Table 1:** Effects of different activation conditions on the interfacial shear strength (IFSS) between the basalt fiber (BF) and epoxy.

Source Gas	Treatment Power (W)	Treatment Pressure (Pa)	Treatment Time (min)	IFSS (MPa)
Control	/	/	/	25.35 ± 1.28
O_2_	100	60	0.5	27.73 ± 1.47
O_2_	200	60	0.5	31.39 ± 1.87
O_2_	300	60	0.5	32.00 ± 2.83
O_2_	200	20	0.5	29.84 ± 1.85
O_2_	200	40	0.5	31.03 ± 2.97
O_2_	200	80	0.5	35.08 ± 1.96
O_2_	200	80	1	34.23 ± 1.76
O_2_	200	80	3	35.21 ± 2.83
O_2_	200	80	5	34.81 ± 3.90
H_2_-Ar	100	80	5	19.46 ± 2.35
H_2_-Ar	200	80	5	24.59 ± 2.50
H_2_-Ar	300	80	5	28.99 ± 2.36
H_2_-Ar	300	20	5	24.82 ± 2.47
H_2_-Ar	300	40	5	26.58 ± 2.18
H_2_-Ar	300	60	5	27.09 ± 2.94
H_2_-Ar	300	80	0.5	25.56 ± 1.57
H_2_-Ar	300	80	1	19.10 ± 2.48
H_2_-Ar	300	80	3	22.02 ± 1.38
H_2_-Ar	300	80	7	27.69 ± 2.04

## Data Availability

The original contributions presented in the study are included in the article, further inquiries can be directed to the corresponding author.
